# Liver Autoimmunity Triggered by Microbial Activation of Natural Killer T Cells

**DOI:** 10.1016/j.chom.2008.03.009

**Published:** 2008-05-15

**Authors:** Jochen Mattner, Paul B. Savage, Patrick Leung, Sabine S. Oertelt, Vivien Wang, Omita Trivedi, Seth T. Scanlon, Krishna Pendem, Luc Teyton, John Hart, William M. Ridgway, Linda S. Wicker, M. Eric Gershwin, Albert Bendelac

**Affiliations:** 1 Howard Hughes Medical Institute, Committee on Immunology, Department of Pathology, University of Chicago, Chicago, IL 60637, USA; 2 Division of Immunobiology, Cincinnati Children's Hospital, Cincinnati, OH 45229, USA; 3 Department of Chemistry and Biochemistry, Brigham Young University, Provo, UT 84602-5700, USA; 4 Division of Rheumatology and Clinical Immunology, School of Medicine, University of California, Davis, Davis, CA 95616, USA; 5 Department of Immunology, The Scripps Research Institute, La Jolla, CA 92037, USA; 6 Division of Rheumatology and Immunology, University of Pittsburgh School of Medicine, Pittsburgh, PA 15261, USA; 7 Juvenile Diabetes Research Foundation/Wellcome Trust Diabetes and Inflammation Laboratory, Department of Medical Genetics, Cambridge Institute for Medical Research, University of Cambridge, Cambridge CB2 0XY, UK

**Keywords:** HUMDISEASE, CELLIMMUNO, MICROBIO

## Abstract

Humans with primary biliary cirrhosis (PBC), a disease characterized by the destruction of small bile ducts, exhibit signature autoantibodies against mitochondrial Pyruvate Dehydrogenase Complex E2 (PDC-E2) that crossreact onto the homologous enzyme of *Novosphingobium aromaticivorans*, an ubiquitous alphaproteobacterium. Here, we show that infection of mice with *N. aromaticivorans* induced signature antibodies against microbial PDC-E2 and its mitochondrial counterpart but also triggered chronic T cell-mediated autoimmunity against small bile ducts. Disease induction required NKT cells, which specifically respond to *N. aromaticivorans* cell wall α-glycuronosylceramides presented by CD1d molecules. Combined with the natural liver tropism of NKT cells, the accumulation of *N. aromaticivorans* in the liver likely explains the liver specificity of destructive responses. Once established, liver disease could be adoptively transferred by T cells independently of NKT cells and microbes, illustrating the importance of early microbial activation of NKT cells in the initiation of autonomous, organ-specific autoimmunity.

## Introduction

Innate immunity is suspected to play a key role in various forms of autoimmunity, but the precise mechanisms involved in activating and propagating autoreactive T and B cells have remained obscure (Benoist and Mathis, 2001; Marshak-Rothstein and Rifkin, 2007). One frequent cause of innate immune activation is represented by overt or unrecognized bacterial or viral infections, but revealing microbial involvement and understanding the cellular and molecular mechanisms leading to autoimmunity generally remain a challenge.

Recent studies in humans have suggested that PBC, an autoimmune disease of the liver, was associated with an antibody response to a particular group of alphaproteobacteria. PBC is a chronic, lethal liver disease characterized by lymphoid infiltration and destruction of small bile ducts leading to bile extravasation and subsequent fibrosis (Kaplan and Gershwin, 2005). It is associated with signature autoantibodies that recognize mitochondrial proteins such as the enzyme PDC-E2, and with autoreactive T cells responding to mitochondrial enzymes (Ueno et al., 2007; Van de Water et al., 1988, 1991; Jones et al., 1995). However, the relationship between antimitochondrial immunity and liver pathogenesis has remained obscure. Recently, molecular mimicry between mitochondria and phylogenetically related alphaproteobacteria such as *Novosphingobium aromaticivorans*, which express conserved PDC-E2 epitopes, has been suggested. *N. aromaticivorans* is a member of the *Sphingomonodaceae* family of Gram-negative alphaproteobacteria, which exhibits xenobiotic-metabolizing properties and is found at mucous surfaces and in the feces of humans (Barbeau et al., 1996; Cavicchioli et al., 1999; Brodie et al., 2007; Pinyakong et al., 2003; Shi et al., 2001; Selmi et al., 2003). Strikingly, patients with PBC expressed antibodies against lipoylated enzymes of *N. aromaticivorans* but not *E. coli* (Kaplan, 2004; Padgett et al., 2005; Selmi et al., 2003).

The cell wall of various strains of *Sphingomonas* (also called *Novosphingobium* or *Sphingobium*), a well-studied genus of the *Sphingomonodaceae* family, is peculiar by its lack of LPS and by the expression instead of glycosphingolipids such as α-glucuronosyl- and α-galacturonosyl-ceramides (Kawahara et al., 2000; Kawahara et al., 1999; Kosako et al., 2000). These unusual glycolipids load CD1d glycoproteins in the lysosomal compartment and are specifically recognized by the conserved, “canonical” T cell receptor of mouse and human CD1d-restricted NKT cells (Figure S1 available online), causing reciprocal activation of NKT cells and dendritic cells and substantial release of Th1 and Th2 cytokines and chemokines (Kinjo et al., 2005; Mattner et al., 2005). Thus, NKT cells and CD1d function as a major innate pathway for the detection of these microbial cell wall components and promote rapid microbial clearance upon infection in vivo. NKT cells accumulate in the liver, where they crawl along sinusoidal endothelial cells, seemingly “patrolling” this vascular space (Ohteki and MacDonald, 1994; Geissmann et al., 2005), and they are increased in the liver of PBC patients (Kita et al., 2002; Harada et al., 2003). Further, increased expression of CD1d was observed in the liver of PBC patients (Tsuneyama et al., 1998). Thus, we hypothesized that NKT cell recognition of *N. aromaticivorans* in the liver might be involved in liver pathology in PBC patients.

We report that mouse infection with *N. aromaticivorans* induced antimitochondrial IgG antibodies and the development of chronic bile duct lesions and lymphoepithelioid granulomas similar to PBC, in a CD1d-dependent manner. Once chronic disease was established, it could be transferred to naive mice by T cells, independently of CD1d, NKT cells, or microbial infection, demonstrating the establishment of an autonomous autoimmune process.

## Results

### *N. aromaticivorans* Infection Model

Infection of mice with various strains of the genus *Sphingomonas* (also termed *Novosphingobium* and *Sphingobium*) results in rapid microbial clearance with usually modest clinical signs of disease (Kinjo et al., 2005; Mattner et al., 2005). To monitor the emergence of PBC, a chronic disease with a long latency period, we inoculated various strains of mice with *N. aromaticivorans* and measured, over several months, autoantibodies to mitochondrial antigens in the serum as well as lymphocyte infiltration and bile duct lesions in the liver. Common mouse strains, including C57BL/6, NOD, and SJL, all exhibited chronic antimitochondrial autoantibodies as well as liver lesions after inoculation of *N. aromaticivorans*. Injection of 5 × 10^7^ cfu intravenously at week 0 and week 2 most consistently induced this chronic disease in nearly all mice. This protocol was therefore used throughout these studies, unless otherwise stated. Furthermore, we focused on the NOD 1101 strain because it exhibited particularly severe enlargement of the liver with lesions suggestive of PBC. NOD1101 belongs to a set of NOD subcongenic strains originating from NOD.c3c4 mice produced by introgressing Insulin-dependent diabetes (*Idd*) loci *Idd*3, 10, 17, and 18 from B6 onto chromosomes 3 and *Idd*9.1-3 from B10 onto chromosome 4 of NOD (Lyons et al., 2000; Wicker et al., 1994). NOD.c3c4 mice do not exhibit diabetes but instead spontaneously develop bile duct lesions and antimitochondrial autoantibodies (Irie et al., 2006; Koarada et al., 2004). NOD 1101 is a subcongenic strain with a restricted B6 chromosomal segment corresponding to *Idd10* and *Idd18* on chromosome 3 (Podolin et al., 1998). Although NOD 1101 mice do not exhibit liver lesions and do not spontaneously develop autoantibody titers, we reasoned that they might harbor some susceptibility genes for bile duct disease.

### *N. aromaticivorans* Infection Induced Long-Lasting IgG and IgA Responses against Mammalian and Microbial PDC-E2

**Figure 1:**
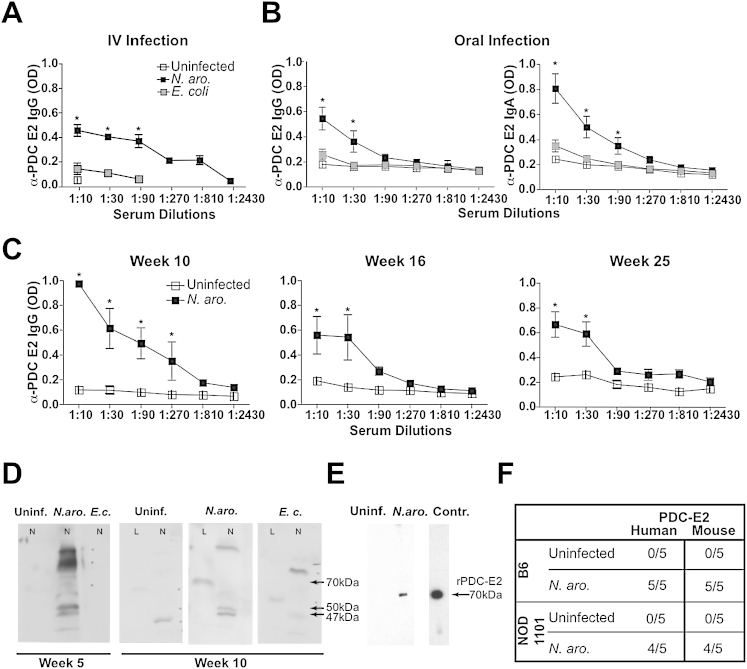
α-PDC-E2 IgG and IgA Responses in Mice Infected with *N. aromaticivorans* (A and B) Mice were inoculated intravenously or orally with *N. aromaticivorans*, *E. coli*, or PBS (uninfected) at week 0 and week 2, and serum IgG and IgA antibodies to recombinant PDC-E2 were measured by ELISA. Results show average and SD values for NOD 1101 mice (three to four mice per group) inoculated with *N. aromaticivorans* or *E. coli* DH5α (5 × 10^7^ cfu intravenously [A] or 1 × 10^9^ cfu orally [B]) and examined at week 10 (left panel) or week 5 (right panels). Data are representative of 20 NOD 1101 mice per group examined in six independent experiments between week 4 and week 12. Similar results were observed when *E. coli* ATCC 25922 was used instead of *E. coli* DH5α. Statistical significance was calculated using a Student's t test: ^∗^p < 0.05 and ^∗∗^p < 0.01. Error bars represent the standard error of the mean for each group. (C) Long-term persistence of anti-PDC-E2 IgG antibodies after infection. Sera of NOD 1101 mice (three per group) were analyzed for the presence of anti-PDC-E2 IgG responses (average and SD) at weeks 10, 16, and 25 as indicated. Error bars represent the standard error of the mean for each group. (D) Western blot analysis of *N. aromaticivorans* (N) or liver (L) lysates using week 5 and week 10 sera from NOD 1101 mice uninfected (uninf.) or inoculated with *N. aromaticivorans* (*N. aro.*) or *E. coli* (*E.c.*), as indicated. Bound antibodies were revealed with mouse IgG-specific secondary antibodies. Note the 47 kDa and 50 kDa bands corresponding to bacterial lipoylated PDC-E2 protein and the crossreactivity to a 70 kDa band in liver extracts corresponding to the molecular weight of mammalian PDC-E2. Results were based on pools of five sera and confirmed in five independent experiments. (E) Western blot analysis of sera from uninfected or *N. aromaticivorans*-infected mice against recombinant PDC-E2. Control (rightmost lane) shows the reactivity of a monoclonal antibody raised against human PDC-E2 (Migliaccio et al., 1998). (F) Presence of antibodies against mouse or human recombinant PDC-E2 in sera of individual B6 and NOD 1101 mice infected with *N. aromaticivorans* (week 7), as detected by western blot (serum dilution 1:500).

**Figure 2:**
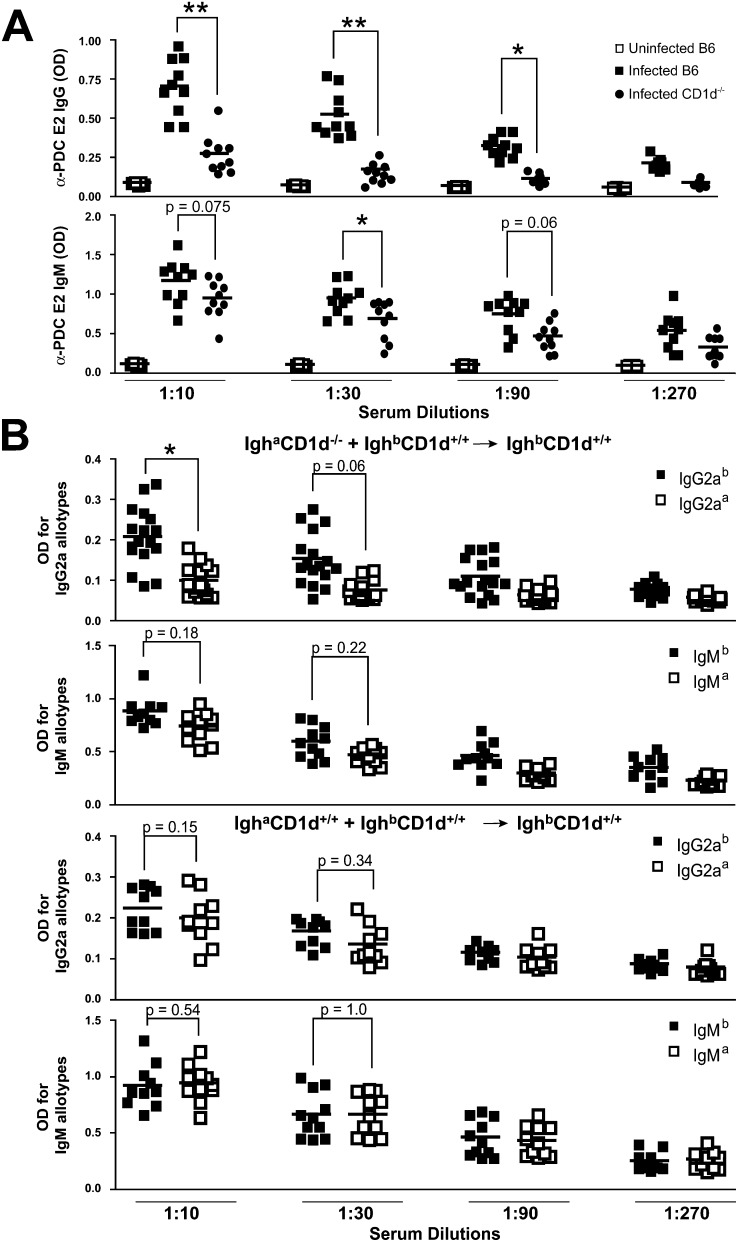
Anti-PDC-E2 IgG Autoantibodies Are CD1d Dependent (A) Wild-type and CD1d-deficient B6 mice (ten per group) were inoculated with *N. aromaticivorans* at week 0 and week 2, and α-PDC-E2 IgG and IgM responses were determined at week 6 in sera diluted as indicated. A cohort of uninfected B6 mice was also analyzed as negative control. Statistical significance was calculated using a Student's t test. A similar result was obtained in another experiment using five mice per group. (B) Irradiated mice reconstituted with a 1:1 mixture of CD1d-deficient Igh^a^ and CD1d-sufficient Igh^b^ bone marrow cells were infected with *N. aromaticivorans* at day 0 and day 14. At day 24, the IgG2a and IgM autoantibodies against PDC-E2 were measured using Igh allotype-specific antibodies to assign their origin to wild-type or CD1d-deficient B cells. Based on FACS staining of B cells for CD1d and for IgM^a^ versus IgM^a^, the chimeras were equivalently reconstituted by the two bone marrows (51 ± 5.8 IgM^a^ versus 54.1 ± 9.2 IgM^b^ and 49.9 ± 6.7 CD1d+ versus 52.3 ± 7.7 CD1d−). Statistical significance was calculated using a Linear-Mixed-Effect Model.

Intravenous inoculation of NOD 1101 with *N. aromaticivorans*, but not *E. coli*, induced an IgG response to PDC-E2, as detected by an isotype-specific ELISA assay of serum samples (Figure 1A). The anti-PDC E2 IgG response included IgG2a (see Figure 2) and also IgG1, IgG2b, and IgG3, whereas little IgA was detected (data not shown). In contrast, IgA autoantibodies were predominantly detected when *N. aromaticivorans* was inoculated orally (Figure 1B). These isotype patterns and the persistence of the IgG response over several months (Figure 1C) differed therefore from the transient IgM and IgG3 autoreactive responses, for example, against anti-nuclear antigens, that have been reported after infection with other bacteria (Fournie et al., 1974; Marshak-Rothstein, 2006; Morrison and Ryan, 1979; Rowley and Jenkin, 1962).

Western blot analysis demonstrated the presence of a 70 kD band in mouse liver and two bands of 47 and 50 kD in *N. aromaticivorans* (Figure 1D) characteristic of PDC-E2 in these different species (Padgett et al., 2005; Selmi et al., 2003). The antibodies induced in B6 as well as in NOD 1101 mice recognized both human and mouse recombinant PDC-E2 (Figures 1E and 1F). These results are consistent with the molecular mimicry hypothesis, suggesting that the autoantibodies are primarily directed against epitopes shared by *N. aromaticivorans* (but not *E. coli*) PDC-E2 and its mammalian homologs (Selmi and Gershwin, 2004).

In addition to PDC-E2, and as also described in patients with PBC, a fraction of mice inoculated with *N. aromaticivorans* expressed IgG autoantibodies against an additional mitochondrial enzyme, BCKD, and against dsDNA (data not shown).

### The Chronic Autoantibody Response Elicited by *N. aromaticivorans* Was CD1d Dependent

Due to the lack of LPS, which is replaced by glycosphingolipid antigens recognized by NKT cells, the major component of innate immunity to the *N. aromaticivorans* cell wall resides with the NKT/CD1d pathway rather than with TLRs (Kinjo et al., 2005; Mattner et al., 2005; Figure S1). We therefore tested whether CD1d was required for the development of autoantibody responses. Since *CD1d* is closely linked to the *Idd*10 locus of NOD 1101, these experiments were performed on a C57BL/6 background where *CD1d*^−/−^ mice are available. Anti-PDC-E2 IgG titers were drastically reduced in CD1d-deficient compared to wild-type mice (Figure 2A), suggesting that NKT cell activation by *N. aromaticivorans* glycosphingolipids provided essential help for the anti-PDC E2 IgG response. IgM antibodies, which can be independent of T cell help, were only modestly decreased (Figure 2A).

NKT cell activation results in systemic release of Th1 and Th2 cytokines, which in turn can broadly enhance distal components of the immune response. Alternatively, it was possible that autoreactive B cells themselves received help from NKT cells, in a direct “cognate” manner, through the simultaneous presentation of *N. aromaticivorans* glycosphingolipid antigens. To test a requirement for cognate NKT-B cell interactions, we reconstituted irradiated BALB/c mice with a mixture of *Igh*^b^ *CD1d*^+/+^, and *Igh*^a^ *CD1d*^−/−^ BALB/c bone marrows. These mixed bone marrow chimeras expressed similar numbers of Igh^a^ (49.9 ± 6.7)- and Igh^b^ (52.3 ± 7.7)-positive B cells as determined by FACS analysis (data not shown). Using allotype-specific reagents, we determined that IgG2a^a^ anti-PDC E2 antibodies were significantly reduced compared with their IgG2a^b^ counterpart when measured 10 days after the second inoculation of *N. aromaticivorans* (Figure 2B, upper left panel). The same pattern was observed 20 days after the second inoculation, although differences did not reach statistical significance (data not shown). In contrast, both allotypes were equally represented at all time points in control chimeras where the two bone marrows expressed CD1d (Figure 2B, upper right panel). By examining IgM production with a similar allotype-specific ELISA assay, we determined that IgM autoantibody production did not require CD1d expression by the secreting B cell (Figure 2B, lower panels). Collectively, and consistent with the well-established helper function of NKT cells (Van Kaer, 2004), these results suggested that IgG2a but not IgM responses depended on NKT cell activation, including cognate interactions between NKT cells and autoantibody-secreting B cells, likely mediated through presentation of microbial glycosphingolipid antigens.

### Chronic Liver Inflammation Induced by *N. aromaticivorans*

**Figure 3:**
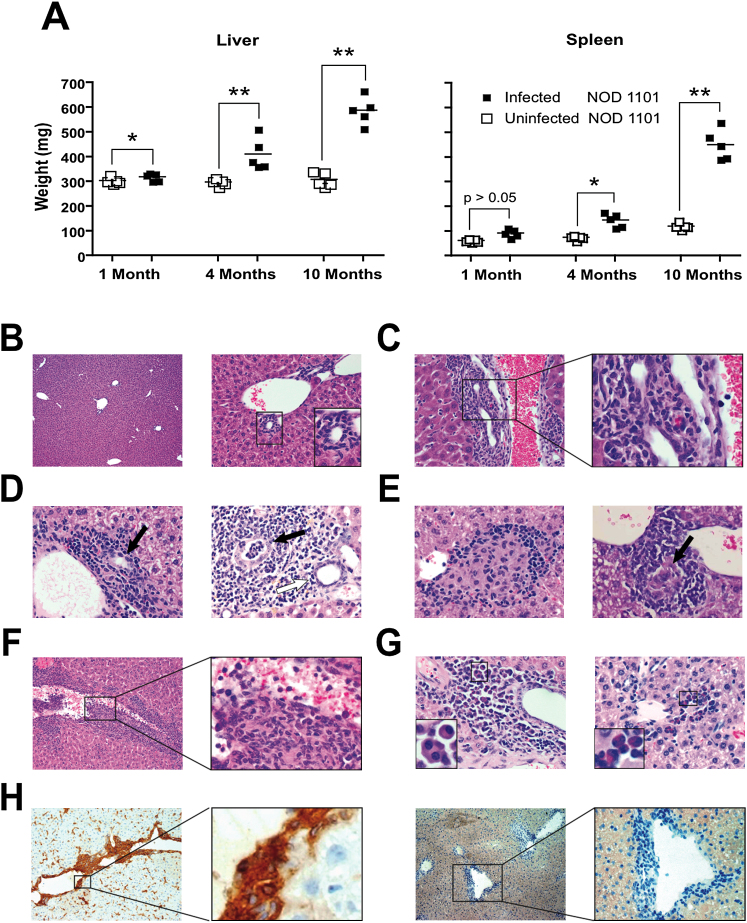
Chronic Liver Disease Induced by *N. aromaticivorans* (A) Progressive increase in liver and spleen weight of NOD 1101 mice at indicated times after *N. aromaticivorans* infection. The results are representative of two independent experiments. Statistical significance was calculated using a Student's t test. (B) Liver sections from an uninfected 7-month-old NOD 1101 mouse. Left panel (5×), right panel (20×), and inset show integrity of bile duct epithelium. (C) Portal inflammation (left panel, 20×) with bile duct damage (right panel, 100×) in a 7-month-old NOD 1101 infected with *N. aromaticivorans* at the age of 1 month. (D) Portal inflammation and bile duct damage in a 4-month-old (left panel, 20×) and 10-month-old (right panel, 20×) NOD 1101 mouse infected with *N. aromaticivorans* at the age of 1 month. In the right panel, note bile duct damage of different intensity (white arrow, bile duct damage with a score of 1; black arrow, bile duct damage with a score of 4). (E) Granuloma with epithelioid cell formation around veins (left panel) and bile ducts (right panel) in NOD1101 mice at 1.5 months (left panel) and 10 months (right panel) postinfection (20×). A damaged bile duct is highlighted by the arrow in the right panel. (F) Venulitis (left panel 10×, enlarged 40× on the right panel) in a 3-month-old NOD1101 mouse infected with *N. aromaticivorans* at the age of 1 month. Note that the inflammatory infiltrate disrupts the vessel wall and that bile ducts cannot be identified on this section. (G) Presence of plasma cells (left panel, 3-month-old B6) and eosinophils (right panel, 10-month-old NOD 1101) (20×). (H) Broad expression of MHC II in a B6 mouse at week 18 after infection (left panel, 10×). Higher magnification shows prominent MHC II expression on bile duct epithelial cells (right panel, 100×); right two panels show the isotype control stainings.

Like other mouse strains (Kinjo et al., 2005; Mattner et al., 2005), NOD 1101 mice cleared most of the bacterial load within a week after infection with *N. aromaticivorans* as judged by CFU analysis of different tissues (data not shown). Further analysis with 16S RNA PCR showed that all tissues, including the liver, were negative by 8 weeks postinfection (see Figures 6C and 6D). These mice, however, progressively developed a massive enlargement of the liver, nearly doubling the organ weight over the course of 10 months (Figure 3A). Other strains, such as SJL, NOD, or B6 (Figure 5A and data not shown) also presented a significant, albeit less important liver enlargement of about 30% over uninfected controls. The spleen became enlarged (Figure 3A) due to lymphocyte accumulation as well as vascular congestion (data not shown). Further, NOD 1101 mice infected at 6 weeks of age failed to thrive and exhibited a lower bodyweight (22.4 ± 8.2 g [n = 5] versus 28.4 ± 3.3 g [n = 5] for uninfected littermates, p < 0.05) at 6 months of age.

Histologic examination of the enlarged livers revealed massive portal inflammation with lymphocyte infiltration and granulomas. In contrast with uninfected littermate controls (Figure 3B), prominent infiltrations of bile ducts (Figure 3C), destruction of biliary epithelial cells with severe bile duct damage (Figures 3C and 3D), and formation of granulomas (Figure 3E) were observed. In some fields, the lymphoid infiltration was so dense that bile duct tissue could not be identified, a finding that was interpreted as a sign of bile duct loss. Lesions of venulitis were also common (Figure 3F). A similar lymphoid infiltration was found in common mouse strains such as SJL, NOD, and C57BL/6 mice, but consistent with their more modest hepatomegaly, these strains seemed to express less severe lymphoid infiltration, although there was some variation depending on the experiments (Figure 5 and data not shown). Severe fibrosis as seen in human PBC was not detected in any of the mouse strains examined. The infiltrates in the portal area were composed of a mixed lympho-monocytic inflammation that contained also plasmocytes and eosinophils (Figure 3G and data not shown), the presence of which is used as diagnostic marker to distinguish PBC from autoimmune hepatitis in humans (Scheuer, 1998; Terasaki et al., 1993). Biliary lesions were accompanied by a persistent upregulation of MHC II on bile duct epithelial cells as well as Kupffer cells, endothelial cells, and inflammatory cells (Figure 3H).

**Figure 4:**
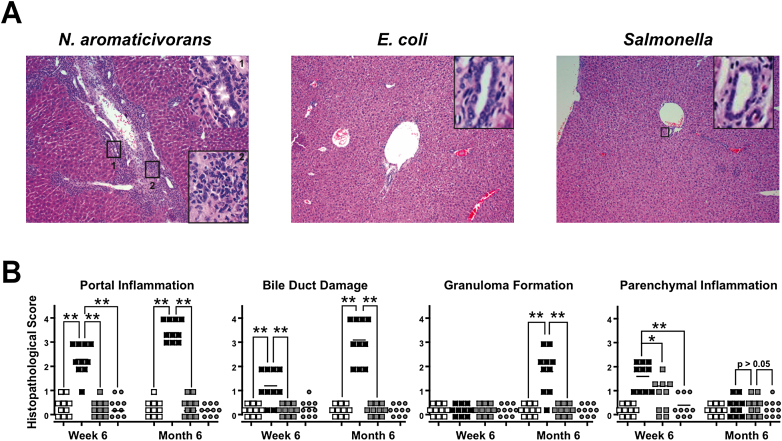
Chronic Liver Disease Is Specific to *N. aromaticivorans* (A) Representative liver sections (10× magnification) of NOD 1101 mice infected with *N. aromaticivorans* (left panel), *E. coli* (middle panel), and *Salmonella* (right panel) 10 months earlier. Note in the left panel the severe portal infiltration with bile duct damage (inset 1, 100×) and moderate fibrosis (inset 2, 100×) after *N. aromaticivorans* infection, contrasting with the integrity of livers infected with *E. coli* or *Salmonella*. (B) Histopathological scores of individual livers at 6 weeks and 6 months postinfection. Statistical significance was calculated using a Mann-Whitney test based on exact p value computations to account for ties. Results are representative of two independent experiments. Mice were infected with 5 × 10^7^*N. aromaticivorans* (filled black squares), 5 × 10^7^*E. coli* (filled gray squares), and 5 × 10^4^*Salmonella* (filled gray circles) cfus, and histopathological scores were analyzed at the indicated time points compared to uninfected control mice (empty black squares).

To evaluate the specificity of these chronic lesions, we examined the livers of mice inoculated with *E. coli* or *Salmonella*. We used a scoring system from 1 to 4 to grade from benign to severe the lesions of portal inflammation, bile duct damage, granuloma formation, and parenchymal inflammation, as described in the Experimental Procedures. While transient inflammation could be detected early after microbial inoculation, as indicated by aggregates of lymphoid and mononuclear cells in the lobular areas or around the periphery of central veins and by hepatocyte necrosis, they resolved within a few weeks, contrasting with the chronic disease after *N. aromaticivorans* infection (Figures 4A and 4B and data not shown). Similar features of transient inflammation have been reported after injection of LPS or α-GalCer alone (Biburger and Tiegs, 2005; Nakagawa et al., 2001; Osman et al., 2000).

Analysis of thyroids, kidneys, joints, and intestines revealed no chronic inflammation in *N. aromaticivorans*-infected NOD 1101 mice (data not shown), further supporting the liver specificity of the inflammation process.

### Chronic Liver Inflammation Depended on CD1d and NKT Cells

**Figure 5:**
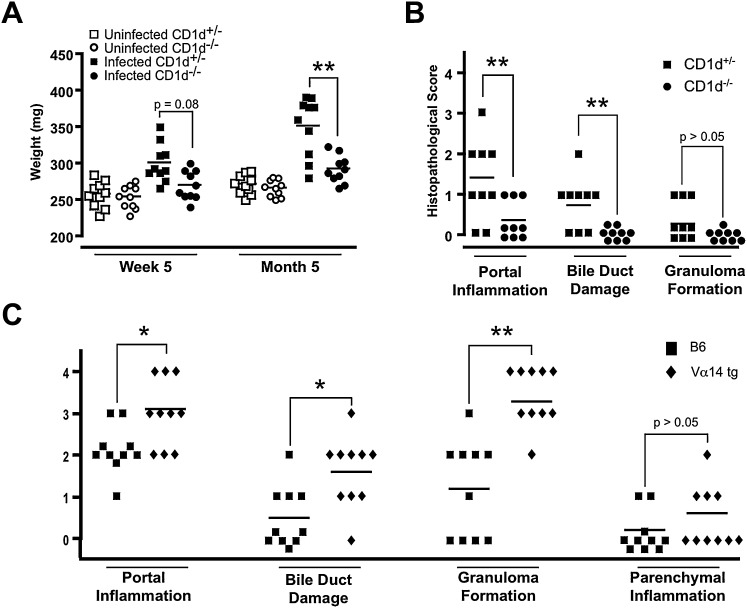
Liver Disease Is Dependent on NKT Cells (A) Liver weights of *CD1d*^+/−^ and *CD1d*^−/−^ B6 littermates at indicated time points after infection with *N. aromaticivorans*. Statistical significance was calculated using a Student's t test. (B) Histopathological score of liver and bile duct lesions of wild-type and CD1d-deficient B6 littermates 4 months after *N. aromaticivorans* infection. Statistical significance was calculated using a Mann-Whitney test. (C) Increased liver pathology in *N. aromaticivorans*-infected B6.Vα14 transgenic compared with wild-type B6 mice. Analysis was performed 2 months after infection, and statistical significance was calculated using a Mann-Whitney test.

To test whether NKT cells were required for chronic liver inflammation, B6 *CD1d*^+/−^ and B6 *CD1d*^−/−^ littermates were infected with *N. aromaticivorans*. Both liver weight increase and histological lesions depended on CD1d (Figures 5A and 5B). Similar results were obtained with *CD1d*^+/−^ and *CD1d*^−/−^ mice on a NOD background (data not shown). Further, B6.Vα14 transgenic mice, which overexpress NKT cells, exhibited more severe histological lesions as well as higher anti-PDC E2 IgG titers than wild-type B6 mice (Figure 5C and data not shown).

### Early but Not Late Antibiotic Treatment Prevented Chronic Liver Inflammation and Autoantibodies

**Figure 6:**
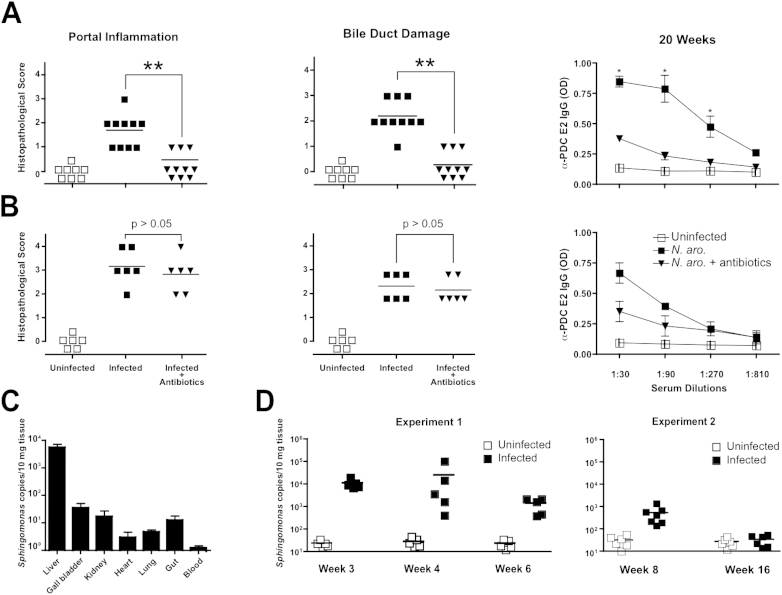
Chronic Disease Is Determined by the Duration of Exposure to *N. aromaticivorans* (A and B) Infected NOD 1101 mice inoculated with *N. aromaticivorans* at week 0 and week 2 were treated with 2 mg/ml Ampicillin and 20 mg/ml Streptomycin in drinking water between weeks 4 and 8 (A) or between weeks 12 and 16 (B). Panels show liver histopathological score for portal inflammation and bile duct damage and serum anti-PDC-E2 antibodies for individual mice at week 20. Groups include uninfected, infected, and infected/antibiotic-treated mice as indicated. Statistical significance was calculated using a Mann-Whitney test (liver lesions) and a Student's t test (antibody response). Error bars represent the standard error of the mean for each group. (C) *N. aromaticivorans* 16S rRNA qPCR in tissues of NOD 1101 mice inoculated at week 0 and week 2 and analyzed at week 3. Average and SD of bacterial copies measured in three individual mice. Error bars represent the standard error of the mean for each group. (D) *N. aromaticivorans* 16S rRNA qPCR in livers of individual NOD 1101 mice inoculated at week 0 and week 2 and analyzed at various time points as indicated.

Chronic liver inflammation and autoantibody production could reflect ongoing responses to persistent microbial infection or, alternatively, represent an immunopathological process evolving autonomously after the initial microbial encounter. We treated one group of mice with a combination of ampicillin and streptomycin between week 4 and 8 (Figure 6A) and another group between week 12 and 16 (Figure 6B). Whereas early treatment abrogated the course of liver disease and the long-term persistence of autoantibodies, late treatment failed to significantly ameliorate disease. Consistent with these results, low bacterial copy numbers could be detected by *N. aromaticivorans*-specific 16S rRNA PCR mainly in liver, but also in gall bladder, gut, and kidney at week 3 (Figure 6C) and up to 8 weeks after infection for the liver (Figure 6D). Although the PCR may amplify other strains in the family of *Sphingomonadaceae*, the low levels detected were well above background as measured in uninfected mice. Collectively, these results suggest that microbial persistence at low copy numbers during the first 4–8 weeks postinfection is required for the late, sterile phase of chronic liver inflammation.

### T Cell Transfer of Liver Disease

**Figure 7:**
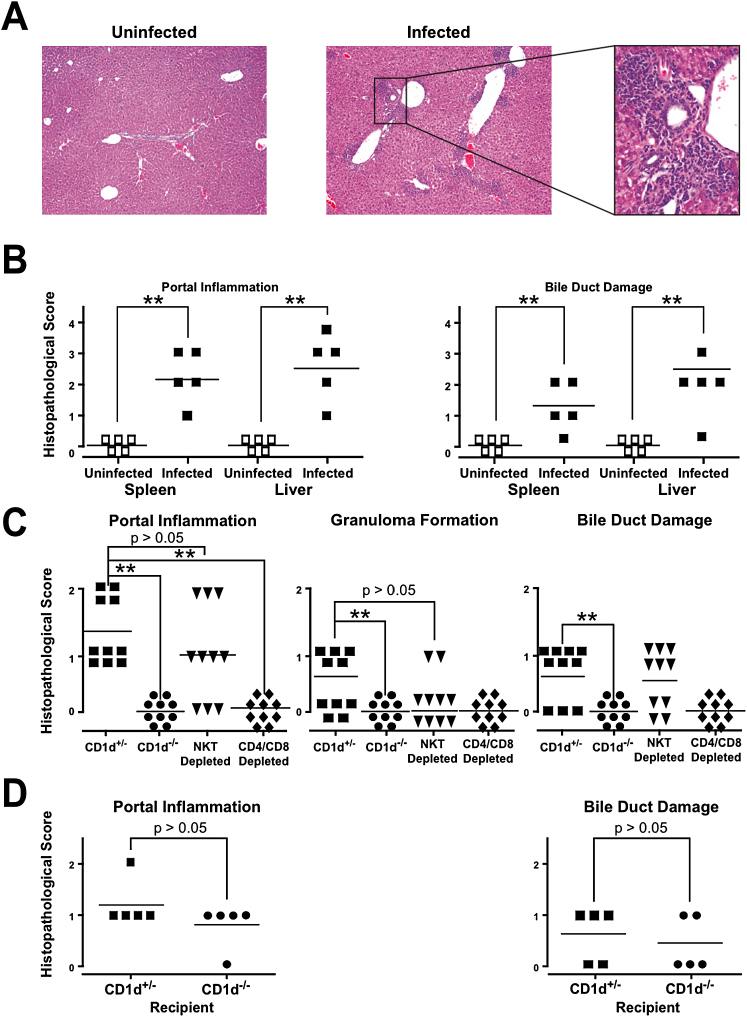
*N. aromaticivorans*-Induced Liver Disease Is Transferred by T Cells (A) Liver sections 6 weeks after syngeneic transfer into adult irradiated recipients of 5 × 10^7^ splenocytes from NOD 1101 donors uninfected or infected 12 weeks before with *N. aromaticivorans*. Note the presence of diffuse lesions in the recipients of splenocytes from infected donors at 10× magnification and the bile duct infiltration at 100× magnification. (B) Histopathological scores of liver lesions after transfer of 2 × 10^7^ splenocytes. Statistical significance was calculated using a Mann-Whitney test. Similar results were obtained after transfer of 5 × 10^6^ liver MNCs (data not shown). These results are representative of two independent experiments with similar results. (C) Transfer of liver disease by 2 × 10^7^ splenocytes of *N. aromaticivorans* -infected B6.*CD1d*^+/−^ but not B6.*CD1d*^−/−^ mice into B6.*scid* recipients. Depletion of CD4+ and CD8+ T cells, but not NKT cells, prevented disease transfer. Results of two independent experiments have been combined, and statistical significance of the differences in histopathological scores was calculated using a Mann-Whitney test. (D) Transfer of splenocytes from *N. aromaticivorans*- infected B6 into irradiated B6.*CD1d*^+/−^ and B6.*CD1d*^−/−^ recipients shows that disease transfer does not require CD1d expression by the host.

The presence of autoantibodies and the evolution toward chronic liver inflammation in the absence of long-term microbial persistence suggested the possibility of an autoimmune origin to the liver disease. We therefore transferred 2 × 10^7^ splenocytes or 5 × 10^6^ liver lymphocytes collected at week 12 postinfection into irradiated (900 Rads) NOD 1101 recipients. Typical liver disease developed within 4–6 weeks after transfer of lymphocytes from infected but not from control, uninfected donors (Figures 7A and 7B). Anti-PDC-E2 antibodies were observed as well (data not shown). As expected from the negative PCR for *N. aromaticivorans* after 8 weeks (Figure 6D), transfer of disease was not modified by the administration of antibiotics to the host and/or recipient (data not shown). To test the role of NKT cells in the transfer of disease, we established a similar transfer system in the B6 background where CD1d-deficient mice are available. Although less severe than in the NOD 1101 background, liver lesions clearly developed in B6.*scid* recipients of spleen cells collected at week 12 postinfection in *CD1d*^+/−^ mice but not *CD1d*^−/−^ mice, confirming the key role of NKT cells in disease (Figure 7C). However, disease transfer did not require NKT cells, as the lesions developed normally in recipients of splenocytes depleted of NKT cells by CD1d-αGC tetramers and MACS. Depletion of CD4 and CD8 T cells, in contrast, abolished the transfer of disease (Figure 7C). Further, liver disease could be induced by T cell transfer into CD1d-deficient as well as CD1d-sufficient recipients (Figure 7D). Collectively, these results imply that NKT cells and CD1d are important to initiate the disease process but that the autoimmune effectors of liver damage are conventional T cells.

## Discussion

A role of *N. aromaticivorans* or related alphaproteobacteria in human PBC was recently suggested based on their phylogenetic relationship with mitochondria, the target organ of PBC patients, particularly the conserved antibody epitope expressed by the enzyme PDC E2 (Kaplan, 2004; Padgett et al., 2005; Selmi et al., 2003). In the current study, we hypothesized that NKT cells, a specialized lymphocyte population that is enriched in the liver might contribute to the pathogenesis of PBC. NKT cells were shown to specifically recognize the cell wall glycosphingolipids of various bacterial strains from the genus *Sphingomonas*, which includes *N. aromaticivorans*, through their semi-invariant CD1d-restricted TCR. By inoculating *N. aromaticivorans* to mice, we established a model of liver disease resembling PBC and we further demonstrated the role of NKT cells in initiating a liver-specific autoimmune process mediated by T cells as well as in helping the production of signature anti-PDC E2 antibodies.

Inoculation of *N. aromaticivorans*, whether intravenously or orally, consistently resulted in the development of chronic liver disease with liver hypertrophy and massive lymphocytic infitration in common strains of mice. Congestive splenomegaly also developed, possibly as an indirect consequence of liver hypertrophy. Key diagnostic elements of PBC were recorded, particularly in the NOD 1101 strain, including the striking infiltration of small bile ducts, their expression of MHC class II molecules, the formation of lympho-epithelioid granulomas, and the presence of eosinophils. Other lesions such as venulitis, hepatocyte lesions, and plasma cell expansion have been more often reported in autoimmune hepatitis, a poorly understood disease entity, or in graft versus host reactions (Czaja, 1994; Scheuer, 1998; Sherlock, 1998; Mieli-Vergani and Vergani, 2004). A notable difference with human PBC, however, was the absence of marked fibrosis in the mouse system. Fibrosis, which is thought to be a tissue reaction to the bile extravasation associated with bile duct epithelium destruction, may involve Th2 cytokines that are suppressed in the mouse system, particularly in the NOD background (Delovitch and Singh, 1997). Indeed, exposure to *N. aromaticivorans* induced relatively little Th2 cytokines from mouse NKT cells in vitro compared with purified glycosphingolipid antigens (data not shown). Collectively, therefore, our findings support the notion that diverse susceptibility or resistance genes influence the final expression of the *N. aromaticivorans*-induced liver disease. Future studies will explore the genetics of *N. aromaticivorans-*induced liver disease in greater detail.

Importantly, liver disease was transferred upon injection of conventional T cells collected from the liver or the spleen of chronically diseased mice, into naive irradiated or SCID syngeneic recipients. While T cell responses to PDC-E2 have been previously demonstrated in PBC patients, consistent with a breakdown of tolerance to this antigen, their pathogenic nature remains uncertain. In contrast, the transfer experiment provided definitive proof of T cell-mediated autoimmunity as a cause of disease, although the autoantigens recognized by disease-causing T cells in this model remain to be elucidated. It is noteworthy that another bile duct disease reminiscent of biliary atresia, which develops in some pediatric patients, was recently observed in mice after rotavirus infection and could be transferred with T cells (Mack et al., 2006).

Completing the cellular response against liver antigens, IgG autoantibodies against PDC-E2, the serological signature of PBC, were observed in all mouse strains examined. We detected anti-dsDNA as well, although at relatively low titer and frequency compared with mouse models of lupus such as NZBxNZW or MRL (data not shown). Nevertheless, it is worth noting that anti-DNA antibodies have also been reported in a fraction of PBC patients and that multiple forms of autoimmunity, including lupus, type I diabetes, Sjogren syndrome, or celiac disease can be found among relatives of some PBC patients (Kaplan and Gershwin, 2005).

NKT cells specifically recognize *N. aromaticivorans* cell wall glycosphingolipids and, in the absence of LPS to activate TLRs, dominate the innate immune response. While they significantly accelerate the clearance of *N. aromaticivorans*, they are not required for recovery. Therefore, their activation upon microbial encounter may be deleterious for the host, providing innate signals that contribute to the breakdown of tolerance and unleash autoimmune effector cells. Indeed, CD1d-deficient mice were largely protected against manifestations of autoimmunity including humoral and cellular responses. Mixed chimera experiments further suggested that NKT cells could directly help CD1d-expressing but not CD1d-deficient anti-PDC-E2 B cells to switch to IgG2a. This finding would imply that B cells recognizing PDC-E2 can capture some glycosphingolipid ligand, presumably attached to the same microbial cell wall fragment to which the enzyme is appended by a lipoyl moiety, for loading onto CD1d in the lysosomal compartment and presentation at the cell surface. Such B cells would then receive NKT cell help, including CD40L and cytokines to induce isotype switch and perhaps somatic hypermutation as well. Consistent with this scenario, CD1d-deficient B cells mainly produced the T helper-independent IgM and IgG3 isotypes.

While NKT cells were clearly required to initiate the cellular and humoral components of disease, they were not necessary at the late chronic phase of the immunopathological process, as evidenced by disease transfer in the absence of NKT cells in the cellular inoculum or in the absence of CD1d in the recipients. This finding supports the notion that NKT cells mainly contribute to the innate phase of the response by provoking the breakdown of tolerance. The expansion of PDC-E2-specific B cells might in turn contribute to the expansion of PDC-E2-specific T cells through efficient surface antibody-mediated uptake of PDC-E2 and subsequent presentation of peptide/MHC complexes (Lin et al., 1991). Other autoreactive B and T cells might be subsequently expanded through a similar process spreading to physically associated or proximal autoantigens (Lehmann et al., 1992; Vanderlugt et al., 1998). In that respect, it is notable that very low levels of *N. aromaticivorans*, as detected by PCR, persisted for several weeks after the bulk of the original microbial inoculum was cleared, perhaps providing continuous fuel for NKT cell activation, and that antibiotic treatment during that period prevented the evolution of chronic cellular and humoral autoimmunity. Consistent with a requirement for prolonged exposure to microbial elements, injection of heat-killed *N. aromaticivorans* induced only transient anti-PDC-E2 response and liver lesions (data not shown). In that context, the preferential activation of NKT cells in the liver, where NKT cells are abundant, crawling along sinusoid endothelial cells and where *N. aromaticivorans* also accumulates, may explain the biased autoreactivity toward autoantigens exposed in the liver environment and, ultimately, the severe organ-specific manifestations of *N. aromaticivorans* infection. The nature of these pathogenic autoantigens, however, remains to be elucidated.

*N. aromaticivorans*, like many other strains of the genus *Sphingomonas* in the *Sphingomonadaceae* family, is an ubiquitous organism found in various marine, terrestrial, and aerosol environments (Barbeau et al., 1996; Cavicchioli et al., 1999; Shi et al., 2001; Brodie et al., 2007). It is also detected by PCR in human feces (Selmi et al., 2003). While this genus only occasionally causes severe infection in immunodeficient individuals (Al-Anazi et al., 2008; Ensminger et al., 2006; Charity and Foukas, 2005), it is likely that most individuals are chronically exposed to these microbial organisms. Our findings raise the question of whether and how aberrant activation of NKT cells might occur in response to this or related organism in the liver of some genetically predisposed patients, ultimately causing or promoting primary biliary cirrhosis. The ability of some strains such as *N. aromaticivorans* to degrade xenobiotic and steroid aromatic compounds may be an important factor (Pinyakong et al., 2003; Shi et al., 2001). Chemical xenobiotics might modify PDC-E2 or other self antigens and lead to loss of tolerance in individuals predisposed to autoimmunity (Gluud, 2002; Amano et al., 2005; Ju, 2005). For example, guinea pigs injected with 6-bromohexanoate developed cholangitis and antimitochondrial antibodies (Leung et al., 2007). Environmental factors have been suggested to induce or exacerbate PBC (Triger, 1980; Uibo and Salupere, 1999; Watson et al., 1995), including urinary tract infections, tobacco, reproductive hormones, and exposure to nail polish. Other studies found increased prevalence of PBC near toxic waste sites (Ala et al., 2006). Based on the mouse infection studies presented here and the previous studies showing crossreactivity of anti-PDC-E2 autoantibodies to *N. aromaticivorans* in humans with PBC, we propose the following conclusions. First, a breakdown of tolerance is a critical component of PBC, as shown by the appearance of autoantibodies against PDC-E2 and the ability of T cells to transfer liver disease. These features also characterize other mouse models of PBC, including the NODc3c4 strain, IL2Rα-deficient mice, and mice transgenically expressing a dominant-negative form of the TGFβ receptor II (Irie et al., 2006; Koarada et al., 2004; Aoki et al., 2006; Wakabayashi et al., 2006; Oertelt et al., 2006). Second, by virtue of coexpressing potent NKT ligands and 2-oxo-dehydrogenase enzymes with high degree of homology to their mitochondrial counterparts, *N. aromaticivorans* has a unique ability to induce PBC. Thus, the present study provides an experimental demonstration and a mechanistic dissection of the role of NKT cells in the transition from early microbially driven immune responses to a stage of autonomous, microbial-independent, T and B cell autoimmunity. These findings warrant further studies on the role of the commensal flora, environmental microbes, and CD1d-restricted NKT cells in human PBC and its different experimental models. We do not suggest that *N. aromaticivorans* is the only possible cause of PBC, but rather that it presents a model that has notable implications for immunological tolerance and autoimmunity.

## Experimental Procedures

### Mice

B6, Balb/c (Igh^a^ and Igh^b^), Balb/c.*CD1d^−/−^*, NOD, SJL, and B6.*scid* mice were purchased from Jackson laboratories. B6.*CD1d^−/−^* mice and the NOD congenic strain 1101 (Podolin et al., 1998) was maintained in our laboratories. The NOD 1101 strain (NOD.B6 I*dd10/18*R2) was obtained from Taconic (Germantown, NY) and is available through the Emerging Models program as Line 1101 (for a description of the introgressed interval, see http://t1dbase.org/cgi-bin/dispatcher.cgi/DrawStrains/display?taconic_line=1101). The NOD 1101 strain was found during the course of this study to harbor a 3.19 Mb B6-derived region on chromosome 18 having a centromeric “out” boundary at rs6303064 (19.74 Mb in Ensembl, NCBI m36) and a telomeric “out” boundary at rs13483251 (22.93 Mb). This region on chromosome 18 is not responsible for the protection from diabetes observed in NOD 1101 mice (Podolin et al., 1998). All mice were MuLV free and were raised in a specific pathogen-free environment at the University of Chicago according to the Institutional Animal Care and Use Committee guidelines.

### Bacterial Strains, Live Infection Experiments, and Treatment with Antibiotics

*N. aromaticivorans* (ATCC 700278), *Salmonella typhimurium* R71, and *E. coli* (DH5α and ATCC 25922) were grown overnight in Mueller Hinton broth, diluted in fresh medium, grown for 8 hr at 37°C to an OD of 0.5 at 600 nm, washed, and diluted in PBS. Bacterial suspension (100 μl) containing 5 × 10^7^ *N. aromaticivorans*, 5 × 10^7^ *E. col*i or 5 × 10^4^ *Salmonella* cfus was injected intravenously into 4- to 20-week-old mice on day 0 and on day 14. Antibiotics were administered in drinking water containing 2 mg/ml Ampicillin and 20 mg/ml Streptomycin.

### Anti-PDC-E2 ELISA

Serum antibodies against the conserved microbial and mammalian PDC-E2 epitope (Padgett et al., 2005) were detected by ELISA against human recombinant PDC-E2 according to the manufacturer's instructions (BioQuant, San Diego, CA), with biotin-conjugated anti-mouse IgG, IgG1, IgG2a, IgG2b, IgG3, IgA, and IgM antibodies obtained from eBioscience (San Diego, CA) or from Southern Biotech (Birmingham, AL).

### Immunoreactivity to Mitochondria and Epitope Specificity Analysis

Serum antibodies to the major mitochondrial antigens PDC-E2, BCOADC-E2, and OGDC-E2 were detected by immunoblotting (with 1:200 and 1:500 serum dilutions) against 20 μg recombinant protein resolved by SDS-PAGE and developed by chemiluminescence as described previously (Padgett et al., 2005; Selmi et al., 2003). A control anti-PDC-E2 mAb (Padgett et al., 2005) was used as a positive control. The specific epitopes recognized were further determined by immunoblotting against the outer lipoyl domain, inner lipoyl domain, E1/E3 binding site, and the catalytic domain of PDC-E2 resolved on 10% SDS-PAGE.

### Immunoreactivity to Microbial and Mammalian Tissue Protein Antigens

Protein lysates from total liver and *N. aromaticivorans* were prepared using a TNE (50 mM Tris-HCl [pH 7.4], 150 mM NaCl, 5 mM EDTA) lysis buffer containing 1 mM Na_3_VO_4_, 1 mM DTT, 5 mg/ml each leupeptin and aprotinin, and 0.5 mM PMSF. Lysate (70 μg) was separated on a 10% SDS page gel and electroblotted to a Polyvinylidene fluoride (PVDF) membrane. Membranes were immunoblotted overnight with 1:100–1:500 dilutions of mouse sera in 5% milk powder followed by a 2 hr incubation with a 1:500 dilution of HRP-conjugated goat-anti-mouse Fab′ peroxidase IgG (Jackson Immunoresearch, West Grove, PA) and detection with ECL Plus (Amersham Pharmacia Biotech, Pittsburgh, PA).

### Mixed Bone Marrow Radiation Chimeras

A mixture of 5 × 10^6^ Igh^b^ *CD1d^+/+^* and 5 × 10^6^ Igh^a^ *CD1d^−/−^* bone marrow cells was injected intravenously into 7- to 12-week-old Igh^b^ *CD1d^+/+^* mice (all on a Balb/c background) that had been 900 Rad irradiated with a cesium source (Gammacell 40, Nordion International Inc. Ontario, Canada) 1 day before. As controls, mixed bone marrow chimeras made with Igh^b^ *CD1d^+/+^* and Igh^a^ *CD1d^+/+^* bone marrow cells were prepared. The B cell reconstitution of the mixed bone marrow chimeras was determined 6–8 weeks after bone marrow injection by FACS analysis of blood samples using anti-IgM^a^, anti-IgM^b^, and anti-CD1d antibodies. The chimeras were intravenously injected with 5 × 10^7^ *N. aromaticivorans* at days 0 and 14, and sera were collected at days 24 and 34. Serum anti-PDC-E2 ELISAs were revealed with the biotinylated, allotype-specific antibodies goat anti-mouse IgG2a^a^, IgG2a^b^, IgM^a^, and IgM^b^ (BD Biosciences, San Diego, CA).

### *N. aromaticivorans*-Specific PCR

Total DNA was extracted from different organs using the DNeasy tissue extraction kit (QIAGEN, Valencia, CA). The following primers were used for detection of *N. aromaticivorans*: forward 5′-TCCGAGTGTAGAGGTGAAAT-3′, reverse 5′-CGTCAATACTTGTCCAGTCA-3′; for control HPRT: forward 5′-ACCTCTCGAAGTGTTGGATA-3′, reverse 5′-CAACAACAAACTTGTCTGGA-3′. Quantitative PCR (qPCR) was performed with 20 μl Brilliant SYBR Green QPCR Master Mix (Stratagene, La Jolla, CA), 1 μl (50 ng) of DNA, 0.5 μl (of 100 nM stock) of each of the forward and reverse primers, and 28 μl of ddH20, using the Mx4000 multiplex quantitative PCR instrument (Stratagene, La Jolla, CA) following the Brilliant SYBR Green QPCR Master Mix manual (Stratagene, La Jolla, CA). *N. aromaticivorans* copy number per 10 mg of tissue was calculated by reference to a standard of pure *N. aromaticivorans* DNA.

### Flow Cytometry

CD1d-lipid tetramers were prepared as described (Benlagha et al., 2000). Anti-B220, -CD19, -CD4, -CD8, -CD3, -TCRβ, -IgM, -IgD, -CD1d, IgM^a^, IgM^b^, and -CD69 antibodies were purchased from PharMingen. Cells were analyzed on a LSR II (BD Biosciences, San Diego, CA) with FlowJo software or a FACSCalibur (BD Biosciences) machine with CellQuest software.

### Cell Transfer Experiments

Splenocytes (2 × 10^7^) or liver MNCs (5 × 10^6^) of diseased (week 12 postinfection) B6.*CD1*^+/−^, B6.*CD1*^−/−^, or NOD 1101 mice were injected i.v. into syngeneic adult B6.*scid* or irradiated NOD 1101 recipients, respectively. Depletion of CD4+ and CD8+ T cells, CD19+ B cells, or CD1d-αGalCer tetramer+ NKT cells was accomplished with the AutoMACs system (Miltenyi Biotec, Germany) using anti-PE or anti-APC beads and following the manufacturer's instructions with purity control by FACS.

### Histology

Liver tissue was fixed in 10% buffered formalin, embedded in paraffin, and cut into 5 μm sections. Liver sections were deparaffinized, stained with hematoxylin and eosin by the University of Chicago Pathology and Histology Research laboratories, and evaluated microscopically in double-blind studies for leukocytic and lymphocytic infiltration. Liver lesions were classified in four different categories (portal inflammation, bile duct damage, granuloma formation, and parenchymal inflammation) and scored by examining 5 sections separated by 25 μM. Portal and parenchymal inflammation were scored using the following scale: 0 = no inflammation, 1 = sparse mononuclear cell infiltrates, 2 = moderate inflammation, 3 = intense inflammation, 4 = intense inflammation and spillover into the periportal parenchyma. The score was based on the most severe infiltration observed in the majority of portal fields or parenchymal areas. Granuloma formation was scored based on the number of granulomas per section: 0 = none, 1 = rare scattered portal and lobular granulomas (<5); 2, 5 to 10 granulomas; 3, 10 to 20 granulomas; 4, more than 20 granulomas. Bile duct damage was scored as follows: 1, epithelial cell hypertrophy, vacuolization, swelling, and flattening could be seen despite the absence of epithelial cell infiltration by lymphocytes; 2, bile duct damage and small infiltration by lymphoid cells; 3, severe bile duct damage and infiltration by lymphocytes; 4, altered bile duct morphology accompanied by massive infiltration by lymphocytes. The infiltration of portal fields was sometimes so dense that it was impossible to identify bile duct epithelium. This could possibly reflect bile duct loss. The bile duct score was based on the most severe lesion observed in at least five bile ducts in one section.

### Immunohistochemistry

Liver sections were embedded in optimal cutting temperature OCT compound (Tissue-Tek, Sakura Finetec, Torrance, CA) and frozen in liquid nitrogen. Five-micrometer-thick sections were cut with a cryostat, placed on 3-amino-propyltriethoxysilane-coated slides, and fixed in ice-cold acetone for 10 min. Slides were incubated in 0.3% H2O2, washed, and blocked with 10% goat serum in Protein Block (Immunicon, Huntingdon Valley, PA). The slides were then incubated with rat anti-CD3 1:100 (BD Biosciences) in a humidity chamber, washed with PBS, and incubated with biotinylated goat anti-rat (Jackson ImmunoResearch Laboratories, West Grove, PA). After washing, the slides were incubated with ABC (Vector Laboratories, Burlingame, CA), washed, and incubated with AEC solution (Scytek, Englewood, CO). The slides were rinsed and counterstained with aqueous hematoxylin blue. Cell subsets were detected with biotin-conjugated antibodies (CD4, CD8, B220, and CD11c cells from eBioSciences; CD11b from Abcam, Cambridge, MA; F4/80 from Serotec, Raleigh, NC). MHC II staining was detected with anti- I-A/I-E 2G9 from BD PharMingen). The slides were then incubated with ABC (Vector Laboratories), washed, incubated with diaminobenzidine (DakoCytomation, Dako, Denmark), and counterstained with hematoxylin blue.

### Statistical Analyses

The tailed Student's t test was used to calculate statistical significance between groups. For Figure 2B, a Linear-Mixed-Effect Model was used. Statistical comparisons of liver histopathological scores were performed using the Mann-Whitney test.

## Supplemental Data

Document S1. Supplemental Experimental Procedures and One Supplemental Figure
