# Supplementary material for: Liver Autoimmunity Triggered by Microbial Activation of Natural Killer T Cells
Source: Cell Host Microbe. 2008 May 15;3(5):304–15. doi: 10.1016/j.chom.2008.03.009 (PMC2453520; doi:10.1016/j.chom.2008.03.009)

Cell Host & Microbe, Volume *3*

Supplemental Data

Liver Autoimmunity Triggered by Microbial Activation of Natural Killer T Cells

Jochen Mattner, Paul B. Savage, Patrick Leung, Sabine S. Oertelt, Vivien Wang, Omita Trivedi, Seth T. Scanlon, Krishna Pendem, Luc Teyton, John Hart, William M. Ridgway, Linda S. Wicker, M. Eric Gershwin, and Albert Bendelac

Supplemental Experimental Procedures

Identification of alpha-glucuronosylceramide (GSL-1) in *Novosphingobium aromaticivorans*

N. aromaticivorans was grown up on TS broth and pelleted via centrifugation. Lipids were extracted and base-labile lipids were removed using the procedure reported by Naka et al. (2000). The crude lipid extract was dissolved in methanol for LC/MS analysis. LC parameters: column-Hypersil SAxNormal phase (50 x 2.1 mm); eluent-ethyl acetate, methanol, water gradient (100% ethyl acetate to ethyl acetate:methanol:water 7:2.7:0.3); flow rate 1 ml/min. MS detection at 352.709, 703.523, 704.531, 721.557, and 726.513 mass units. Agilent LC/MSD (TOF).

NKT cell hybridoma stimulation assays

Bone marrow-derived DCs were prepared in RPMI/10 % FCS supplemented with rmGM-CSF and rmIL- 4 (R&D Systems, Minneapolis, MN) as described (Mattner et al., 2005). 100,000 DCs/well were pulsed for 8 hours with titrated doses of heat killed or live *N. aromaticivorans* or synthetic GSL-1 and exposed to 100,000 DN32.D3 NKT hybridoma cells. Cells were co-cultured in 96-well round-bottom plates for 24 hours and cell culture supernatants were assayed for IL- 2 release by ELISA (R&D Systems, lower detection limit of 15 pg/ml).

Supplemental References

Chiu, Y.H., Park, S.H., Benlagha K., Forestier C., Jayawardena-Wolf J., Savage P.B., Teyton L., and Bendelac A. (2002). Multiple defects in antigen presentation and T cell development by mice expressing cytoplasmic tail – truncated CD1d. Nat. Immunol., *3*, 55-60

Naka, T., Fujiwara, N., Yabuuchi, E., Doe, M., Kobayashi, K., Kato, Y., and Yano, I. (2000) A novel sphingoglycolipid containing galacturonic acid and 2-hydroxy fatty acid in cellular lipids of *Sphingomonas yanoikuyae*. J. Bacteriol., *182,* 2660-2663.

Mattner, J., Debord, K. L., Ismail, N., Goff, R. D., Cantu, C., 3rd, Zhou, D., Saint-Mezard, P., Wang, V., Gao, Y., Yin, N.*, et al.* (2005). Exogenous and endogenous glycolipid antigens activate NKT cells during microbial infections. Nature *434*, 525-529.

Figure S1. *N. aromaticivorans*-derived alpha-glucuronosylceramide (GSL-1) stimulates NKT cells.
(A and B) Characterization of alpha-glucuronosylceramide (GSL-1) by liquid chromatography and mass spectrometry. A, Liquid chromatograms of synthetic GSL-1 (left panel) and GSL-1 isolated from *N. aromaticivorans* (right panel). B, Mass spectra of synthetic GSL-1 (left panel) and GSL-1 isolated from *N. aromaticivorans* (right panel). Ions at 704 and 726 mass units correspond to $[M+H]^+$ and $[M+Na]^+$, respectively.

(C and D) NKT cell stimulation by N. aromaticivorans alpha-glucuronosylceramide. Dendritic cells from wild type B6, CD1d-deficient B6 ($CD1^{-/-}$), or tail-deleted CD1d (TD) B6 (Chiu et al., 2002) were pulsed for 8 hours with heat-killed (C, left panel) or live (C, right panel) *N. aromaticivorans* at the indicated bacteria:DC ratios or with synthetic GSL-1 (D) at the indicated concentrations and exposed to the NKT cell hybridoma DN32.D3. The release of IL-2 was measured 24 hours later using ELISA and the results of two independent experiments were combined. Levels of IL-2 release in unstimulated samples were below the detection limit of the ELISA (not shown). Error bars represent the standard error of the mean for each group.

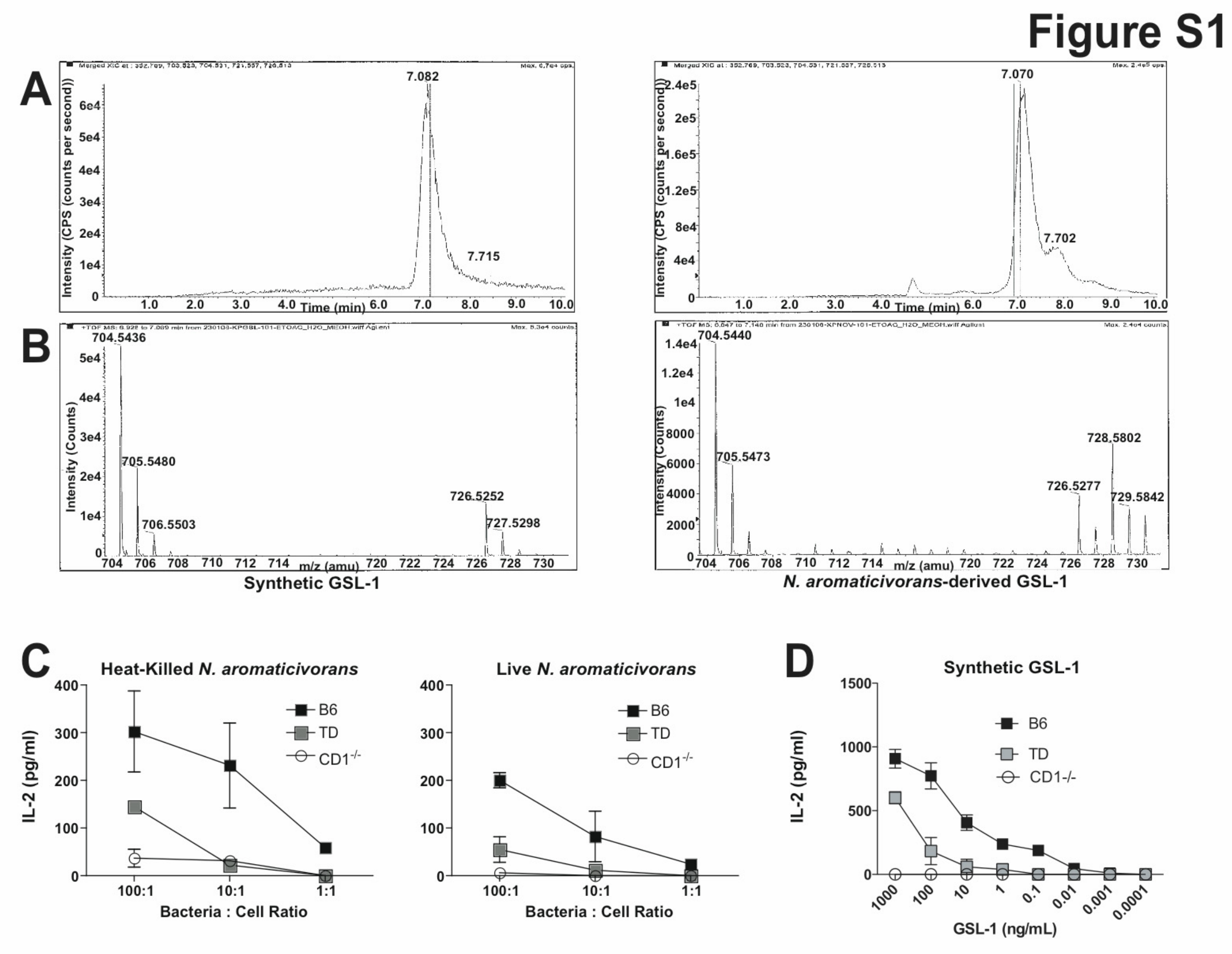

Supplement: Document S1. Supplemental Experimental Procedures and One Supplemental Figure [file mmc1.pdf]
